# Unintentional firearm deaths in the United States 2005–2015

**DOI:** 10.1186/s40621-019-0220-0

**Published:** 2019-10-14

**Authors:** Sara J. Solnick, David Hemenway

**Affiliations:** 1 Department of Economics, University of Vermont, 239 Old Mill, 94 University Place, Burlington, VT 05405 USA; 2 Harvard T.H. Chan School of Public Health, 677 Huntington Avenue, Boston, MA 02115 USA

**Keywords:** Gun, Firearm, Unintentional, Deaths, Fatalities

## Abstract

**Background:**

Unintentional gun death occurs four times more often in the United States than other high-income countries. Research on these deaths typically has a narrow scope. We believe this is the first study describing the circumstances of these deaths in the United States that covers more than a single state or municipality.

**Methods:**

We use data on all unintentional firearm fatalities in the sixteen states reporting to the National Violent Death Reporting System (NVDRS) for all years 2005–2015. Our final count of unintentional firearm deaths in these states and years is 1260. The detailed nature of the data allows us to categorize and compare the circumstances of the incident.

**Results:**

We estimate 430 unintentional firearm fatalities in the United States per year. The rate is highest for older children to young adults, ages 10 to 29, and the vast majority of the victims are male. Common circumstances include playing with the gun (28.3% of incidents), thinking the gun was unloaded (17.2%), and hunting (13.8%). The victim is suspected to have consumed alcohol in nearly a quarter of the deaths and in 46.8% of deaths among those aged 20–29.

**Conclusions:**

Certain circumstances, such as consuming alcohol, playing with the gun, and hunting, are common settings for unintentional firearm deaths. Firearm safety instructors, firearm manufacturers, and firearm owners can all contribute to preventing these deaths.

## Background

Unintentional firearm deaths decreased in the United States for all ages from 2000 to 2012 (Fowler et al. 2015; Griffin et al. 2018) and among children from 2002 to 2014 (Fowler et al. 2017). Nevertheless, unintentional deaths comprise 9% of firearm deaths around the world (The Global Burden of Disease 2016 Injury Collaborators, 2018). While that study of 195 countries showed that the rate of unintentional firearm deaths in the United States is the same as that for the 195 countries combined, the unintentional firearm death rate in the United States is four times higher than that of the other high-income countries (Grinshteyn and Hemenway 2019).

Despite the scale of the problem, there has been little attention to establishing basic information about victims, perpetrator (self or other), and circumstances. Just as with firearm deaths generally, unintentional firearm deaths comprise many different scenarios. In order to devise effective policy, it is essential to understand the various circumstances that can lead to a fatal outcome, because different interventions targeted at different pathways may be necessary to reduce the toll.

Few prior studies have examined the circumstances of unintentional firearm injuries, and the characteristics of the sample population; key findings of this literature are summarized in Table 1 (Copeland 1984; Morrow and Hudson 1986; Ornehult and Eriksson 1987; Martin et al. 1991; Harruff 1991; Cherry et al. 2001; Karger et al. 2002; Junuzovic and Eriksson 2012; Junuzovic et al. 2016). These studies typically have a sample size below 100, with some samples falling between 200 and 400 cases. Because sample sizes are small, the most robust findings relate to the percentage of deaths in two specific circumstances: hunting and playing with the gun. In these nine papers, the proportion of unintentional firearm deaths from hunting varies greatly by location, from 0% in a study of the unintentional firearm deaths of 25 New Mexico children (Martin et al. 1991) to 59% of unintentional firearm deaths in Sweden over thirteen years (Ornehult and Eriksson 1987).

**Table 1:** Summary of prior research on circumstances of unintentional firearm fatalities

Year	Title & Authors	Location	Ages	Time period	N	% Hunting	% Playing with gun
1984	Accidental death by gunshot wound — fact or fiction Copeland AR	Metro-Dade county, FL, USA	all	1972–1982	54	7%	22%
1987	Accidental firearm fatalities during hunting Ornehult L, Eriksson A	Sweden	all	1970–1982	47	59%	–
1986	Accidental firearm fatalities in North Carolina, 1976–80 Morrow PL, Hudson P	North Carolina, USA	all	1976–1980	210	19%	19%
1991	Accidental firearm fatalities among New Mexico children Martin JR, Sklar DP, McFeeley P	New Mexico, USA	0–14	1984–1988	25	0%	68%
1992	So-called accidental firearm fatalities in children and teenagers in Tennessee, 1961–1988 Harruff RC	Tennessee, USA	0–19	1961–1988	225	17%	44%
2001	A population based study of unintentional firearm fatalities Cherry D, Runyan C, Butts J	North Carolina, USA	all	1985–1994	390	10%	35%
2002	Accidental firearm fatalities. Forensic and preventive implications Karger B, Billeb E, Koops E	Two cities in Germany	all	1967–1997	32	19%	19%
2012	Unintentional firearm hunting deaths in Sweden Junuzovic M, Eriksson A	Sweden	all	1983–2008	48	53%	–
2016	Unintentional Nonhunting Firearm Deaths in Sweden, 1983–2012 Junuzovic M, Sjoberg A, Eriksson A	Sweden	all	1983–2012	43	–	30%

The percentage of deaths caused by playing with a gun ranges from 19 to 35% in all-ages samples. In a study of 0–19 year olds, 46% of the unintentional gun deaths took place while playing with a gun (Harruff 1992), and in a study of 0–14 year olds, more than two-thirds of the deaths were attributed to playing with a gun (Martin et al. 1991).

Other research on unintentional firearm deaths has sought to describe the source of the weapon or the identity of the shooter. Research that focused on children found that unintentional fatalities were often caused by a gun in the victim’s home or the home of a friend or relative (Grossman et al. 1999) and the shooter was most often a friend or family member, usually male and also a child (Hemenway and Solnick 2015).

Finally, another branch of research investigates the effect of gun prevalence and firearm storage practices and policies on unintentional injuries at the population level (Wiebe 2003, Grossman et al. 2005; Miller et al. 2005; Hepburn et al. 2006; Miller et al. 2002a; Miller et al. 2002b; Faulkenberry and Schaechter 2015; Santaella-Tenorio et al. 2016; Levine and McKnight 2017; Hamilton et al. 2018).

In this paper, we provide a detailed descriptive epidemiology of unintentional firearm fatalities in the United States. Using data from the National Violent Death Reporting System (NVDRS) over eleven years, we are able to categorize and compare the circumstances of the incidents. We believe this is the first study describing the circumstances of these deaths in the United States that covers more than a single state or municipality.

## Methods

We examine all unintentional firearm fatalities in states reporting to the NVDRS for all years 2005–2015. Twenty-four states participated during that time, but eight of them (Arizona, Connecticut, Kansas, Maine, Michigan, Minnesota, New York, and Ohio) did not begin contributing until 2010 or later and were excluded. The sixteen states that reported for all years in our time period are Alaska, Colorado, Georgia, Kentucky, Maryland, Massachusetts, New Jersey, New Mexico, North Carolina, Oklahoma, Oregon, Rhode Island, South Carolina, Utah, Virginia, and Wisconsin. These states had 26.6% of the United States population during that time. These states also had 26.8% of all firearm deaths in the United States in that time period, based on Vital Statistics data compiled by the Centers for Disease Control and Prevention (CDC).

Our data on unintentional firearm fatalities comes from the NVDRS which is a state-based surveillance system that links data from the death certificate, law enforcement records, and coroner/medical examiner records on deaths due to suicide, homicide, legal intervention, unintentional firearm injury, and firearm fatalities of unknown intent (Paulozzi et al. 2004, Hemenway et al. 2009, Barber et al. 2013). The system does not generate new data, but combines data that were already being collected. Reporting is considered comprehensive (i.e., all deaths in the categories are included) but deaths that are misclassified, such as a suicide that is attributed to a pre-existing medical condition, will not be captured (Weiss et al. 2006). However, the system is designed so that every firearm death should be included in the NVDRS.

NVDRS records are incident-based and include information on the persons (victims and perpetrators), weapons, and circumstances involved. In addition to the coded data, the abstractor writes incident narratives summarizing the findings from both the coroner/medical examiner records and the law enforcement records. Data are collected and linked at the state level, stripped of personal identifiers, and forwarded to the Centers for Disease Control and Prevention. Data for this study come for the NVDRS Restricted Access Dataset. The study was deemed exempt by the Harvard T.H. Chan Human Subjects Institutional Review Board since it uses only non-identifiable publicly available data.

The participating NVDRS states were not selected to be nationally representative (Blair et al. 2016), nor does the CDC provide weights that can be employed to extrapolate the data to reflect the United States population. In order to develop a rough national estimate of unintentional gun deaths, we assume that the number of unintentional firearm fatalities in the sixteen NVDRS states is in proportion to the population or to the number of all firearm fatalities of those states. We then scale up the number of deaths in the NVDRS using both assumptions to develop a rough national estimate.

The NVDRS places unintentional firearm deaths into three categories: self-inflicted, inflicted by other person, and unknown who inflicted (11% of cases). The authors of this paper reviewed every violent death report that was classified as any type of unintentional firearm death (*N* = 1289). We read both the medical examiner and police narratives for all of them. Based on these descriptions, we re-categorized 29 cases from an unintentional firearm death to an intentional (suicide or homicide) death or a non-firearm death. Another 74 cases remained as unintentional firearm deaths but the perpetrator was re-classified from self to other or from unknown to self or to other. Our final count of unintentional firearm deaths in these states and years is 1260. We did not read the many cases categorized as suicides or homicides by the abstractors to determine if any might be more correctly classified as unintentional, so counts may be considered minimums. However, compared to Vital Statistics which often miscategorizes unintentional firearm deaths to children as homicide and undetermined firearm deaths to adults as unintentional, NVDRS has been shown to be highly accurate in classifying unintentional firearm fatalities (Barber and Hemenway 2011).

We further categorize these deaths by determining the activities the perpetrator was involved in at the time, and whether the event was alcohol-involved. For information on the circumstances of the shooting, certain variables are already classified in the data. These include whether the incident involved hunting (which includes the period preparing for the hunt and restoring the firearms following the hunt as well as during the hunt itself), whether the perpetrator had been playing with the gun, whether the perpetrator thought the gun was unloaded, whether the incident took place during loading or cleaning of the gun, and whether the victim was suspected of alcohol consumption. For other circumstances—specifically whether the hunt was for large game or small game and whether the injury occurred when the person holding the gun fell or dropped the weapon—we classified incidents based on the description in the narratives. The categories are not mutually exclusive. For example, a death could occur while hunting and when the victim is suspected of using alcohol and would be included in the counts for both circumstances. In addition, there were fatalities that did not fall into any of the circumstances, often because of insufficient detail in the narrative (or lack of any narrative).

## Results

Over the eleven years and sixteen states in our study, we found 1260 unintentional gun fatalities in the NVDRS data (Table 2). Using the percentage of the United States population in these states during that time, we perform a “back-of-the-envelope” calculation and estimate that the national number of unintentional gun fatalities would be 4741 over eleven years or 431 per year. An alternative calculation is based on the percentage of all firearm deaths in the United States that took place in these states in that time period, based on Vital Statistics data compiled by the Centers for Disease Control and Prevention. Using that percentage, we estimate that the national number of unintentional gun fatalities would be 4696 over eleven years or 427 per year.

**Table 2:** Unintentional firearm deaths (16 states, 2005 to 2015): Basic demographics by victim age (*N* = 1260)

	Victim Age							
	0–9	10–19	20–29	30–39	40–49	50–59	60+	ALL
Population in the 16 states 2005–2015	117,917,508	123,237,820	124,723,170	119,910,860	128,953,694	122,913,548	165,197,724	902,854,324
All unintentional								
Number	138	306	257	110	140	120	189	1260
Rate per million	1.2	2.5	2.1	0.92	1.1	0.98	1.1	1.4
Self-inflicted deaths								
Number	63	97	134	63	85	73	127	642
Rate per million	0.53	0.79	1.1	0.53	0.66	0.59	0.77	0.71
Percent of deaths	52.1%	34.0%	57.5%	64.3%	69.1%	72.3%	80.9%	57.4%
Percent male victim	81.0%	93.8%	94.8%	93.7%	88.2%	95.9%	94.5%	92.4%
Other-inflicted deaths								
Number	58	188	99	35	38	28	30	476
Rate per million	0.49	1.5	0.79	0.29	0.29	0.23	0.18	0.53
Percent of deaths	47.9%	66.0%	42.5%	35.7%	30.9%	27.7%	19.1%	42.6%
Percent male victim	65.6%	84.6%	81.8%	82.9%	73.7%	75.0%	76.7%	79.6%

Age is an important factor in the rate and type (self-inflicted versus other-inflicted) of unintentional firearm death (Table 2). The groups at highest risk are older children and teens, ages 10 to 19, with a rate of 2.5 per million population, and young adults, aged 20 to 29, with a rate of 2.1 per million population. All other age groups have a markedly lower rate, ranging between 1.4 and 0.9 per million population.

Among the older children and teens (ages 10 to 19), other-inflicted deaths are more common, comprising 66.0% of the unintentional firearm deaths. For every other age group, self-inflicted deaths are more common, and the percentage increases from 57.7% for the youngest adults (20 to 29 years old) to 80.9% for the oldest group (aged 60 or older).

The vast majority of the victims are male. For self-inflicted deaths, the youngest age group, 0 to 9 years old, has the lowest percentage of male victims at 81.0%. Males are 88.2% of victims for 40 to 49 year-olds and males are above 93% of victims for every other age group. For other-inflicted deaths, a strong majority of victims are male. Again, the youngest age group stands out, being only 65.6% male. In every other age group, more than 73% of the victims of other-inflicted deaths are male. For every age group, female form a larger percentage of the victims of other-inflicted deaths than of self-inflicted deaths. Males are overwhelmingly the shooters in unintentional firearm deaths. When they are shooting someone other than themselves, they occasionally shoot a female.

Unintentional gun deaths can be characterized by the circumstances of the incident (Table 3). Playing with the gun is the most common scenario, especially for younger victims. A majority of unintentional firearms deaths for children ages 0 to 9 occur when they or someone else are playing with the gun. The percentage of incidents in which the perpetrator was playing with the gun falls from 63.8% for the youngest group, to 42.8% for older children/teens (age 10 to 19), to 36.6% for young adults (age 20–29), and to 24.6% for those aged 30 to 39. For victims aged 40 and older, it is uncommon for someone to be killed while playing with a gun, with the percentage ranging from 1.7 to 4.3%.

**Table 3:** Unintentional firearm deaths (16 states, 2005 to 2015): Percentage of cases with specific circumstances by victim age (*N* = 1260)

	Victim Age							
	0–9	10–19	20–29	30–39	40–49	50–59	60+	ALL
Playing with gun^a^	63.8%	42.8%	36.6%	24.6%	4.3%	1.7%	4.2%	28.3%
Thought unloaded^a^	10.1%	26.8%	24.5%	18.2%	6.4%	8.3%	10.1%	17.2%
Hunting^a^	3.6%	14.7%	9.3%	15.5%	16.4%	22.5%	17.5%	13.8%
Big game^b^	0.7%	6.5%	4.7%	8.2%	5.7%	5.0%	4.8%	5.2%
Small game^b^	1.5%	2.9%	1.6%	0.9%	3.6%	4.2%	7.9%	3.3%
Unclassified^b^	1.5%	6.9%	3.9%	6.4%	7.9%	14.2%	5.8%	6.3%
Loading or cleaning^a^	5.1%	13.1%	13.2%	10.9%	14.3%	20.0%	17.5%	13.5%
Fell or dropped gun^b^	7.3%	4.9%	3.5%	6.4%	7.1%	10.0%	7.9%	6.2%
Suspected alcohol^a^	0.0%	14.9%	46.8%	38.6%	27.5%	14.1%	11.3%	22.7%

^a^These variables are predefined in the NVDRS

^b^These variables were defined by searching for key words and then reviewing the narratives

Another common scenario for unintentional death is when someone thought the weapon was unloaded. This is most common for victims aged 10 to 39, with the frequency ranging from 26.8% for ages 10 to 19 to 18.2% for ages 30 to 39. For other age groups, this situation occurs 10% of the time or less.

For all ages, some unintentional firearm deaths occur while hunting, which includes setting out for and returning from the hunt as well as during the hunt itself. The percentage is smallest for the youngest age group (0 to 9 years old), at 3.6%. For all other age groups, hunting is the context for between 9.3 and 22.5% of unintentional firearm deaths. For ages 10 to 59, the hunts leading to unintentional firearm deaths are more likely to be for big game, such as deer or moose, than for small game, such as rabbits, ducks, or squirrels. Small game hunts are more common in the unintentional firearm deaths of the youngest and oldest age groups (ages 0 to 9 and age 60 and older). Unintentional death can happen when someone is loading or cleaning the firearm. This cause is less frequent for children under 10 years old (5.1% of incidents), is more common for those aged 10 to 49 (between 10.9 and 14.3% of incidents) and is most frequent in the older age groups, aged 50 and older (between 17.5 and 20.0% of incidents).

Fatalities sometimes occur when someone falls or drops a weapon. This situation has a fairly low probability for all ages of victims, ranging from a high of 10.0% for victims aged 50 to 59 to a low of 3.5% for victims aged 20 to 29.

Nearly a quarter of cases involve alcohol consumption. Alcohol was not suspected to be a factor for the deaths of the youngest group (ages 0 to 9), but alcohol was thought to be present in 14.9% of the cases for 10 to 19 year-olds. The rate of alcohol involvement is highest for the 20 to 29 year olds, at 46.8% and then declines steadily to 11.3% for the oldest group (aged 60 or older).

## Discussion

A study using death certificate data from the National Center for Health Statistics from 1999 to 2006 found 574 unintentional firearm deaths for adults (Carr et al. 2012). Our lower rate of approximately 430 per year may be due to more careful classification of unintentional firearm deaths. The NVDRS has been shown to more accurately categorize unintentional firearm deaths than Vital Statistics (Barber and Hemenway 2011).

Two studies of unintentional firearm deaths while hunting conducted in Sweden (which has fewer guns per capita and stronger gun laws than the United States) divided the incidents into small game and big game hunting. In the earlier time period, 1970 to 1982, small game hunting accounted for 62% of deaths and 38% of deaths occurred during moose hunting (Ornehult and Eriksson 1987). In a more recent study, which covers 1983 to 2008, small game hunting accounted for 54% of the deaths and moose hunting 46% of the total (Junuzovic and Eriksson 2012). Following the Swedish studies, we divided hunting into small- and big-game hunting. We found that the proportion of small game hunting to big game hunting deaths varied by age, with small game hunting deaths more common for the youngest and oldest groups (ages 0 to 9 and 60 and older) and big game hunting deaths more common for victims ages 10 to 59.

Previous studies of the United States show that the percentage of deaths that occur while playing with the gun varies by age. The two studies of children found high percentages of incidents that took place while someone was playing with the gun: 64% of deaths in the study of children 14 and younger in New Mexico (Martin et al. 1991) and 44% of deaths in a study of victims aged 19 and younger in Tennessee (Harruff 1992). The studies that included all ages had lower proportions of deaths due to playing with the gun, ranging from 35% (Cherry et al. 2001) to 19% (Morrow and Hudson 1986; Karger et al. 2002). In our data, playing with the gun was the context in 63.8% of the incidents for the youngest group (ages 0 to 9) and 42.8% for the older children and teens (ages 10 to 19), with the percentage falling to under 4.5% for victims ages 40 and older.

The rates of unintentional firearm fatalities, and the types, vary across states. Not surprisingly, unintentional firearm fatality rates vary across states with the level of household gun ownership (Miller et al. 2001), and hunting deaths vary with rates of hunting. Our set of states includes those with low rates of gun ownership (e.g., MA, NJ, RI) and states with high rates (e.g., Alaska, Kentucky, Oklahoma). Rates of hunting licenses also range from less than 1.5 per 100,000 (MA, NJ, RI) to more than 10.5 per 100 (AK, KY, OK, OR, WI) (Siegel et al. 2014).

This study has limitations. First, it provides data for only 16 states (for eleven years). However, while the states were not randomly selected, the group includes states that are urban and rural, east and west coast, northern and southern, high-gun and low-gun. These states contain 26.6% of the United States population and had 26.8% of the gun deaths from all causes. The gold standard measure of state-level household gun ownership rates is CDC’s annual Behavioral Risk Factor Surveillance System (BRFSS) (Azrael et al. 2004; Okoro et al. 2005; Siegel et al. 2014). Using the most recent responses on this question from the BRFSS, which date from 2004, the sixteen states in our analysis had an overall household gun ownership rate of 33%; the other 34 states had an overall household gun ownership rate of 31%. Thus, the states in our data have a similar percentage of households with firearms as the other 34 states. The NVDRS is expanding to cover all 50 states which will greatly increase its usefulness.

Second, while most cases were accompanied by one or two narratives that provided useful qualitative data, for some there were no narratives, or the ones provided did not provide much information--such as on how the gun was accessed or exactly what happened. For example, in the cases classified as “hunting;” there were many cases for which we could not determine whether the hunters were pursuing big game or small game. Nor do we know how often circumstances (e.g., cleaning; thought unloaded) were missed or misreported. We suspect that rates for most of the categorizations in this paper should be thought of as underestimates.

Third, although this study includes several times more cases than previous work, certain categories remain uncommon. For this reason, we combined incidents in which someone was loading or cleaning a firearm and we combined incidents in which someone fell or dropped a gun. Any confidence intervals for many estimates would be wide (e.g., comparing the percentage of unintentional firearm deaths that are hunting-related for different age groups), especially for categories determined from reading the short narratives.

## Conclusion

This study provides valuable information about the actual number and circumstances of unintentional firearm deaths in the United States. Unintentional shootings kill approximately 430 Americans every year and almost all these tragic deaths are preventable. Victims span the age spectrum but are most likely to be older children, teens and young adults (ages 10 to 29), and the vast majority of both victims and shooters are male. Understanding the various circumstances that lead to unintentional fatalities is an essential step to address the problem. Consuming alcohol, playing with the gun, and hunting, are common settings for these deaths. Firearm safety instructors, firearm manufacturers, and firearm owners can all contribute to reducing the death toll.

## Data Availability

The data that support the findings of this study are available from Centers for Disease Control and Prevention but restrictions apply to the availability of these data, which were used under license for the current study, and so are not publicly available.
